# Risk-taking behavior in juvenile myoclonic epilepsy

**DOI:** 10.1111/epi.12413

**Published:** 2013-10-18

**Authors:** Britta Wandschneider, Maria Centeno, Christian Vollmar, Jason Stretton, Jonathan O’Muircheartaigh, Pamela J Thompson, Veena Kumari, Mark Symms, Gareth J Barker, John S Duncan, Mark P Richardson, Matthias J Koepp

**Affiliations:** * Department of Clinical and Experimental Epilepsy, UCL Institute of NeurologyLondon, United Kingdom; † Epilepsy Society MRI Unit, Epilepsy SocietyChalfont St Peter, United Kingdom; ‡ Department of Neurology, Epilepsy Center, University of MunichMunich, Germany; § Department of Neuroimaging Institute of Psychiatry, King’s College LondonLondon, United Kingdom; ¶ Department of Psychology Institute of Psychiatry, King’s College LondonLondon, United Kingdom; # Department ofClinical Neurosciences, Institute of Psychiatry, King’s College LondonLondon, United Kingdom

**Keywords:** Impulsivity, Frontal lobe function, Functional imaging, Working memory

## Abstract

**Objective:**

Patients with juvenile myoclonic epilepsy (JME) often present with risk-taking behavior, suggestive of frontal lobe dysfunction. Recent studies confirm functional and microstructural changes within the frontal lobes in JME. This study aimed at characterizing decision-making behavior in JME and its neuronal correlates using functional magnetic resonance imaging (fMRI).

**Methods:**

We investigated impulsivity in 21 JME patients and 11 controls using the Iowa Gambling Task (IGT), which measures decision making under ambiguity. Performance on the IGT was correlated with activation patterns during an fMRI working memory task.

**Results:**

Both patients and controls learned throughout the task. Post hoc analysis revealed a greater proportion of patients with seizures than seizure-free patients having difficulties in advantageous decision making, but no difference in performance between seizure-free patients and controls. Functional imaging of working memory networks showed that overall poor IGT performance was associated with an increased activation in the dorsolateral prefrontal cortex (DLPFC) in JME patients. Impaired learning during the task and ongoing seizures were associated with bilateral medial prefrontal cortex (PFC) and presupplementary motor area, right superior frontal gyrus, and left DLPFC activation.

**Significance:**

Our study provides evidence that patients with JME and ongoing seizures learn significantly less from previous experience. Interictal dysfunction within “normal” working memory networks, specifically, within the DLPFC and medial PFC structures, may affect their ability to learn.

Juvenile myoclonic epilepsy (JME) is the most common idiopathic epilepsy syndrome (Berg et al., 2010). Patients have been described as “immature” with impaired experience-related learning (Janz, 1985; De Araujo Filho & Yacubian, 2013), suggestive of frontal lobe dysfunction. This is corroborated by reports on impaired working memory (WM) and executive functions (Wandschneider et al., 2012) and functional and microstructural changes within the medial and dorsolateral prefrontal cortex (DLPFC) (Vollmar et al., 2011; O’Muircheartaigh et al., 2011).

The Iowa Gambling Task (IGT) was developed for patients with lesions within the prefrontal cortex (PFC) presenting with impulsive decision making irrespective of possible future consequences (Bechara et al., 1994). Previous studies investigated the relationship of decision making with performance in other cognitive domains of the PFC, such as executive functions (EFs) and working memory (WM), arguing for a dissociation of decision-making abilities from other cognitive prefrontal lobe functions (Toplak et al., 2010). However, there is also evidence for an asymmetrical relationship of decision making and WM in healthy subjects (Suhr & Hammers, 2010) and in patients with frontal lobe lesions (Bechara et al., 1998; Manes et al., 2002), but also in nonlesional frontal lobe disorders (Duarte et al., 2012). Working memory performance may not depend on intact decision-making abilities, but WM dysfunction can mediate impaired decision making. Lesions or dysfunction of the anterior ventromedial prefrontal cortex (VMPFC) and orbitofrontal cortex seem to be associated with impaired decision making only, whereas pathology in the posterior (VMPFC) is associated with both WM and decision making impairment and the DLPFC with WM impairment only (Bechara et al., 1998; Brand et al., 2007).

These observations are corroborated by a recent functional MRI (fMRI) study investigating neuronal correlates of decision making during the IGT in healthy controls (Li et al., 2010).

Cortical activation patterns underlying decision making include the following: (1) the DLPFC for WM, in order to provide online knowledge during the decision-making process; (2) insula and posterior cingulate for emotional feedback; (3) medial orbitofrontal cortex to interlink the previous two processes; and (4) ventral striatum and supplementary motor area (SMA) to carry out decisions (Li et al., 2010). Impulsive decision making in the IGT has been reported recently in JME (Zamarian et al., 2013), and was specifically evident if seizures were poorly controlled.

We aimed to investigate the underlying mechanisms of decision-making behavior in JME, in particular to elucidate which part of the WM network, as defined by fMRI, is associated with IGT performance (Li et al., 2010).

## Methods

This study was approved by the UCL Hospital Research Ethics Committee. Written informed consent was obtained from all study participants.

Twenty-one JME patients and 11 healthy controls (HC), matched for gender (JME: 12 female, HC: 6; Pearson chi-square p = 0.888), age (JME: median 33.5 [range: 22–64] years; HC: 28 [24–32]; Mann-Whitney U = 70.500, N_1_ = 21, N_2_ = 11, p = 0.074), and IQ (JME: median 109 [range: 77–118]; HC: 113 [107–116]; Mann-Whitney U = 51.000, N_1_ = 20, N_2_ = 7, p = 0.314) were assessed with the IGT. With regard to educational exposure, 10 patients were postgraduate, two obtained A-levels, six General Certificate of Secondary Education (GCSE), and in three patients, information about educational exposure could not be obtained. Performance was correlated with activations during an fMRI WM task (n-back). Imaging data sets from two controls and two patients were excluded because of lack of activations during fMRI. In one patient and two HCs, we could not record the dot back performance due to joystick failure.

Patients had a typical history of JME and had been diagnosed by experienced epileptologists according to the International League Against Epilepsy (ILAE) Classification (Berg et al., 2010). All clinical MRI scans were normal on qualitative analysis. Fourteen patients had been seizure-free for at least 12 months (see Table S1 for further clinical details). No patient experienced a seizure during the assessment.

### Standardized frontal lobe tests

A battery of standardized tests assessing frontal lobe functions was administered to all participants. It included the following:

1. Letter and category fluency. The letters “F,” “A,” and “S” were presented for letter fluency, and the categories “animals,” “fruits,” and “vegetables” for categorical fluency. The number of words produced for each letter or category within one minute was totaled to provide an average score for letters and categories.
2. The Digit Span subtest of the Wechsler Adult Intelligence Scale (WAIS-III; Wechsler, 1981) for working memory;
3. The Trail Making Test (Reitan & Wolfson, 1985) for
  1. Psychomotor speed: Total time to complete part A.
  2. Mental flexibility: A score was derived by subtracting the time to complete for part A from the time to complete part B.

### Iowa Gambling Task

In a computerized IGT version (Bechara, 2007), subjects chose from four decks of cards (A–D) with a total of 100 cards. All decks awarded virtual monetary gains and losses. The aim was to gain as much money as possible. Decks A and B were associated with higher gains and penalties, indicative of disadvantageous choices. Decks C and D were associated with lower gains and penalties (advantageous choices).

Before commencing the IGT, participants were instructed that their aim was to gain as much money as possible and to avoid losing money. However, they were not informed about the IGT’s contingencies and had to learn them by feedback from their card choices, that is, their monetary gains and losses.

#### Scores of IGT performance

1. The total number of card selections from the disadvantageous decks (A and B) and the total number of selections from the advantageous decks (C and D) were counted. Subsequently, an overall net score was derived by subtracting the total number of disadvantageous from advantageous choices (C + D − [A + B]). Net scores above zero indicate that subjects select advantageously; net scores below zero indicate disadvantageous choices.
2. The same score as in (1) was derived for five consecutive blocks of 20 cards. The IGT mainly measures decision making under ambiguity, as especially initial choices are made without explicit knowledge of their consequences and decisions are mainly guided by implicit information. However, it has been shown that healthy subjects optimize their performance during the task by learning the properties of each deck and subsequently change their strategy toward choosing from the advantageous decks only (Brand et al., 2007). By obtaining net scores for five consecutive blocks we aimed at characterizing changes in IGT performance and strategy over time (Brand et al., 2007; Li et al., 2010).
3. In addition, for each individual, changes in task performance indicative of experience-related learning throughout the task were analyzed. Similarly to Li et al. (2010), we subtracted the net score of the first block from the net score of the fifth block. A score above zero was indicative of a positive slope in the individual learning curve. A score of zero or below was indicative of a negative or unchanged slope in the learning curve. Accordingly, participants were divided into learners and nonlearners.

### Statistical analysis

Data were analyzed using SPSS Statistics Version 17.0 (SPSS Inc., Chicago, IL, U.S.A.). Group differences were analyzed with statistical tests for nonparametric data. Chi-square tests were applied to categorical data.

### Functional MRI paradigm

MRI data were acquired on a GE Excite HDx 3T scanner (GE Medical Systems, Milwaukee, WI, U.S.A) with a multichannel head coil as described previously (Vollmar et al., 2011). The WM paradigm consisted of dots presented randomly in four possible locations on a screen (Kumari et al., 2009). Participants monitor the locations of dots at a given delay of the original occurrence (0-, 1-, or 2-back) and respond by moving a joystick corresponding to the location of the current or previously presented dot. FMRI analysis was performed using SPM-5 (www.fil.ion.ucl.ac.uk/spm). For each subject, contrast images were created to explore the effect of “2-back minus 0-back” comparing the task with the highest WM load (“2-back”) against the control task; hence by controlling for motor response and visual attention, only cortical activation due to the WM load was revealed.

#### Correlation analyses

To test for correlations between IGT performance and network activations during the fMRI task, a whole brain multiple regression analysis was performed and overall net scores of IGT performance were entered as a covariate.

We explored the effect of seizures on IGT performance at group level by creating a full factorial analysis of covariance with seizures (seizure-free/seizures) as a factor. Within the above analysis, a global conjunction analysis was performed to explore the effect of overall IGT performance between groups (HC, seizure-free patients, and patients with ongoing seizures).

We further explored the effect of seizures on learning throughout the IGT in a subgroup analysis of patients by creating a full factorial analysis with seizures (seizure-free/seizures) and learning (learner/nonlearner) as factors.

The level for significance was p < 0.005 uncorrected.

## Results

### Behavioral data

#### Performance on standardized frontal lobe test battery

The results are detailed in Table 1. There were no significant group differences in performance on the frontal lobe test battery. Because learning to shift strategy toward more advantageous card choices may rely on executive functions (Brand et al., 2007), we conducted a subgroup analysis of frontal lobe measures in the patient groups of nonlearners versus learners, which showed no significant differences (see Table S2).

**Table 1:** Neuropsychological test results

Cognitive abilities	Controls	Patients	Statistical analysis^a^	
	Median (range)	Median (range)	U	p
Psychomotor speed				
Trail Making Test A (time in seconds)	36 (21–41)	28 (18–50)	42.000	0.130
Mental flexibility				
Trail Making Test time B-A (time in seconds)	31 (7–44)	32 (5–62)	58.500	0.533
Verbal fluency				
Categorial fluency	19.0 (17.33–22.0)	19.17 (12.33–24.0)	65.500	0.808
Letter fluency	14.33 (11.0–19.66)	14.66 (9.0–21.66)	76.000	0.862
Working memory				
Subtest Digit Span of the WAIS-III	19 (14–23)	19 (9–28)	42.000	0.731

a The Mann-Whitney U test was applied for behavioral measures.

#### IGT performance

No significant differences in the total net scores as index of advantageous choices were observed (JME: median 7 [range: −50 to 68]; HC: 28 [range: −4 to 58]; Mann-Whitney U = 107.500, N_1_ = 21, N_2_ = 11, p = 0.755).

When analyzing the net scores over the five consecutive blocks, both groups learned throughout the task (last versus first block: Wilcoxon test, JME: z = −3.163, N-Ties = 20, p = 0.002; HC: z = −2.347, N-Ties = 10, p = 0.019).

**Figure 1:**
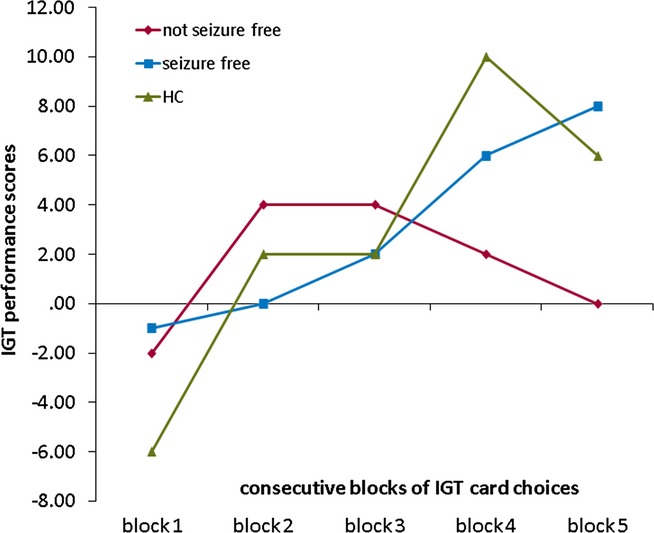
IGT performance over time in seizure-free and not seizure-free patients, as well as in controls. Scores in patients and healthy controls over five consecutive blocks of 20 IGT card choices: Positive scores represent more advantageous choices, and negative scores represent more disadvantageous choices. Compared to not seizure-free patients, controls and seizure-free patients shifted their choices toward more advantageous selections continuously throughout the task. HC, healthy controls; IGT, Iowa Gambling Task.

Analyzing changes in IGT performance throughout the task in each participant showed that the majority of patients and controls are learners (JME: 15 learner/6 nonlearner; HC: 8/3; Pearson chi-square p = 0.938). In a post hoc subgroup analysis, the majority of patients who were seizure-free improved throughout the task (12 learner/2 nonlearner), whereas only half of the patients still experiencing seizures changed their strategy toward more advantageous choices over time (3 learner/4 nonlearner; Pearson chi-square p = 0.040; Fig. 1).

### fMRI findings

Performance on the fMRI WM task was comparable for both groups with a median 71% success rate (range: 23–100) in patients and 90 (43–100) in controls (Mann-Whitney U = 48.500, N_1_ = 19, N_2_ = 8, p = 0.147). A subgroup analysis comparing performance on the fMRI WM task in patients with ongoing seizures to seizure-free patients identified no significant group differences (seizure-free patients: median 69% (range: 23–100); patients with ongoing seizures: 73 (52–98); Mann-Whitney U = 34.500, N_1_ = 13, N_2_ = 6, p = 0.701).

In a further subgroup analysis, IGT performance expressed as the overall net score did not correlate with the performance on the fMRI WM task in either group: seizure-free patients r = 0.456, p = 0.117; patients with ongoing seizures r = −0.512, p = 0.299; healthy controls r = 0.481, p = 0.114.

**Figure 2:**
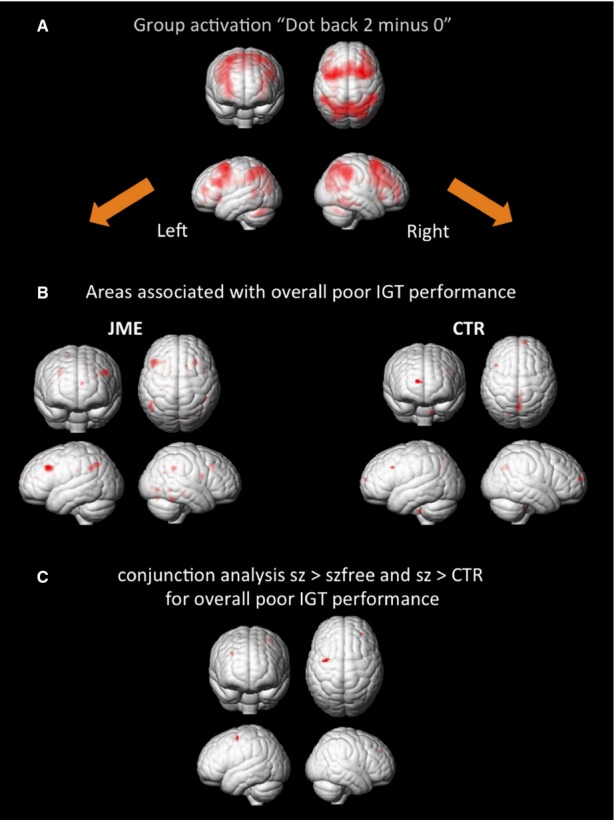
Association of working memory network activation with overall IGT performance. (**A**) Both groups, JME patients and controls, activate bilateral frontal and parietal working memory networks during the working memory task (“2-back minus 0-back”-contrast). (**B**) Overall net scores of IGT performance (C + D − [A + B]) were entered as a covariate. Negative correlation of IGTperformance with the “2-back minus 0-back” contrast across the whole JME group revealed bilateral prefrontal cortex activation. (**C**) Conjunction analysis of JME patients with seizures above seizure-free patients and controls, and negative correlation with overall IGT performance revealed hyperactivity in the left DLPFC. The contrast was masked by working memory network activations of healthy controls (p < 0.05 unc.) (CTR, controls; IGT, Iowa Gambling Task; JME, juvenile myoclonic epilepsy; sz, seizures; szfree, seizure-free).

The “2-back minus 0-back” contrast showed significant bilateral frontal and parietal WM network activations (Fig. 2A).

In patients, poor IGT performance was associated with bilateral PFC activation. In controls, poor performance was associated with reduced deactivation in parts of the default mode network, that is, in the bilateral precuneus, cingulate gyrus, and right medial PFC (Fig. 2B). In a conjunction analysis of patients with ongoing seizures compared to both healthy controls and seizure-free patients, patients with seizures showed increased activity in the left DLPFC in association with poor IGT performance (Fig. 2C).

Using WM performance scores as an additional covariate of no interest in the fMRI regression analysis, similar neuronal association patterns were identified (Fig. 1, Supporting Information).

**Figure 3:**
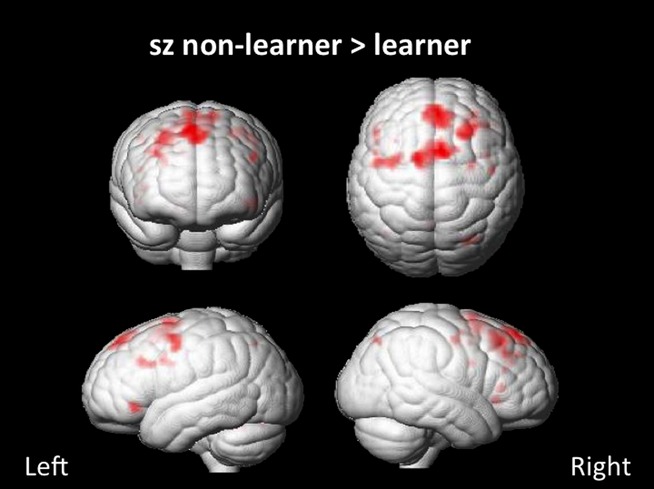
Association of working memory network activation with learning in patients. A subgroup analysis in patients with ongoing seizures showed a bilateral medial prefrontal cortex and pre-SMA, a left dorsolateral prefrontal cortex (DLPFC), and right SFG activation in nonlearners compared to learners. (p < 0.005 unc.) (sz, seizures).

Within the group of patients still experiencing seizures, nonlearners showed greater activations in bilateral medial PFC and pre-SMA, as well as the left DLPFC and right superior frontal gyrus (SFG) when compared to learners (Fig. 3).

## Discussion

We report network abnormalities associated with decision-making behaviors in patients with JME. We identified impaired IGT performance only in the group of JME patients still having seizures, which is similar to a previous study reporting continuing impairment in experience-related learning in patients with ongoing seizures compared to those without (Zamarian et al., 2013). Because both patient subgroups and controls performed equally well in the WM fMRI task, it is possible to relate cortical activation patterns with IGT performance. In JME, overall poor IGT performance was associated with bilateral DLPFC activation. Ongoing seizures and overall poor IGT-performance were associated with increased left DLPFC activation.

### Overall IGT performance

Patients with ongoing seizures and seizure-free patients were comparable in their performance during the WM fMRI task. Patients and controls did not differ significantly in the behavioral measures of cognitive functions. This may be in keeping with reports that decision making is relatively independent from other frontal lobe functions, such as WM (Toplak et al., 2010). However, in a bigger cohort, Zamarian et al. (2013) could show that patients with JME performed significantly worse on overall IGT net scores. Similarly, when comparing seizure-free patients to those with ongoing seizures in our cohort, JME patients with ongoing seizures showed severe impairment of their IGT performance (Fig. 1).

Our fMRI findings suggest that crucial hubs within physiologic WM networks are implicated in impaired IGT task performance in patients. Studies in healthy subjects (Suhr & Hammers, 2010) and patients with nonlesional frontal lobe disorders (Duarte et al., 2012) describe an asymmetrical relationship between WM and IGT performance, in the sense that WM dysfunction may moderate decision-making impairment. The DLPFC is recruited during continuous updating of WM and has been identified as part of the neuronal circuitry underlying decision making both in fMRI studies in healthy individuals (Li et al., 2010) and lesional studies (Manes et al., 2002). In our cohort, left DLPFC activation was associated with impaired decision making only in patients with ongoing seizures.

Previous behavioral studies reported subtle working memory impairment in JME patients (Wandschneider et al., 2012). Causative brain pathology for frontal lobe dysfunction is not apparent on clinical MRI scans. However, there is evidence for microstructural abnormalities (O’Muircheartaigh et al., 2012), suggesting a thalamo-frontocortical network dysfunction and increased functional and structural connectivity between cognitive and motor networks (Vollmar et al., 2011, 2012), which correlate with disease activity. These network changes are present even in high functioning JME patients with no WM impairment on behavioral measures (Vollmar et al., 2011; O’Muircheartaigh et al., 2011). Therefore, DLPFC hyperactivation may be the neuroanatomic correlate of a seizure-related network dysfunction, implicating WM networks, and through this moderating impaired decision making. DLPFC hyperactivation has been seen in other patient groups with low WM capacity, such as patients with schizophrenia (Manoach, 2003), and may also reflect a necessary compensatory mechanism of cortical activation to achieve the WM performance required for the task.

In controls, poor IGT performance was associated with decreased deactivation in the default mode (DM) during the active condition. DM areas are independent of task active areas, and suspension of baseline DM brain function allows reallocating neuronal resources to task-relevant brain areas. Inability to deactivate the DM during the active phase of tasks has been generally related to poor performance during the active task condition (Raichle et al., 2001) and may thus be the explanation for its relation to poor IGT performance in the control cohort. Similarly, in a cohort of patient with temporal lobe epilepsy and HC, a failure to deactivate parts of the DM network has been related to poor performance during the same WM fMRI task employed in our study (Stretton et al., 2012). More specifically, for decision making, decreased deactivation in areas of the DM may imply that the process of learning probabilities of future events is impaired. This is corroborated by a recent study investigating decision making under uncertainty in healthy subjects (D’Acremont et al., 2013); the authors employed a gambling task to investigate how subjects learn probabilities of future events irrespective of their (immediate) value. The stage of learning during the task was associated with progressive deactivation of DM, including the angular gyrus, medial PFC, and posterior cingulate cortex.

### Learning during the IGT

The effects of ongoing seizures and impaired learning throughout the task were associated with bilateral medial prefrontal cortex (PFC) and pre-SMA, right SFG, and left DLPFC activation. Brand et al. (2007) argued that the IGT comprises two phases: during the first trials, subjects learn to make choices from implicit information; during the second phase, subjects have acquired some explicit knowledge about the IGT contingencies, hence choices are made under risk, that is, with the knowledge of future consequences. The switch between the phases probably happens gradually, and the latter phase is more dependent on the integrity of the DLPFC and frontostriatal loops, as making successful choices under risk requires executive functions, such as set shifting (Brand et al., 2007). Similarly, in an event-related fMRI study, Lawrence et al. (2009) identified prefrontal subregions contributing to different stages of the IGT. In healthy individuals, activation in posterior frontal regions and the pre-SMA were positively correlated with general task performance. In terms of neuronal correlates of learning, the ventrolateral PFC and the pre-SMA were recruited early during the task, when subjects were acquiring knowledge about the contingencies. With more explicit awareness of task strategy and reduced uncertainty, a corresponding decrease in pre-SMA activation was observed in good performers. Accordingly, other studies identified the pre-SMA as an area involved in guidance of behavior in uncertainty situations and decision making under uncertainty (Ridderinkhof et al., 2004; Hartstra et al., 2010). In our cohort, pre-SMA activation is seen in patients with ongoing seizures who do not learn to shift their IGT strategy. This may imply that nonlearners do not gain explicit awareness about task strategies and continue to make choices under ambiguity; therefore, they do not show a decrease of pre-SMA activation over time.

## Limitations

In terms of the possible impact of AED treatment on IGT performance, 8 patients were treated with monotherapy, 11 with combination therapy (10 patients with two AEDs, one patient with three), and one patient did not receive any AED. The majority of patients were on valproic acid (VPA; 13 patients), followed by levetiracetam (LEV; 9) and lamotrigine (LTG; 7). Generally, AEDs may affect cognitive abilities, and cognitive side effects vary among different agents and are more prominent with combination therapy (Hermann et al., 2010). Processing speed and attention are particularly vulnerable domains, however, these did not appear to be affected in our sample when comparing patients to healthy controls. LTG and LEV have been both associated with rather little negative effect on cognition (Hermann et al., 2010). For LTG, beneficial effects on frontal lobe functions have been described due to its mood-stabilizing properties (Pavuluri et al., 2010). One study in JME patients (Roebling et al., 2009) reported a negative effect of VPA treatment on a verbal memory test when compared to healthy controls. A previous fMRI study in our cohort, however, reported a dose-dependent normalizing effect of VPA on abnormal working memory network activation with motor-cortex coactivation (Vollmar et al., 2011). Because of the small subgroup sample sizes, specific AED effects on cognition could not be explored in more detail. As there was no difference in behavioral measures between groups, and the majority of patients was treated with AEDs, and the distribution of monotherapy versus polytherapy was equal in learners and nonlearners (see Table S1), we suggest that poor IGT performance is at least in part independent of AED treatment.

## Conclusions

Our results add further evidence that ongoing seizure activity affects daily life decision making, and emphasize the importance to achieve full seizure control. Learning and IGT performance were impaired, especially in JME patients with ongoing seizures. Learning and nonlearning patients did not differ in behavioral frontal lobe measures, but did differ in IGT performance and neuronal network correlates within the DLPFC and thalamo-frontocortical loops. These structures are important for successful implementation of executive functions during decision making under risk (Brand et al., 2007), and the ventromedial PFC is crucial for overall successful task performance and learning to win (Lawrence et al., 2009). Our findings illustrate that neuropsychological deficits may be too subtle to detect on conventional behavioral measures, and underscore the importance of imaging studies and more challenging frontal lobe tasks, such as the IGT, which integrates decision-making behavior and executive functions, to detect differences in cognitive networks.

## Supporting Information

**Figure S1.** Association of working memory networks activation with IGT performance.

**Table S1.** Clinical details.

**Table S2.** Neuropsychological test results in patients (learners vs. nonlearners).
