# Supplementary figures and images for: Risk-taking behavior in juvenile myoclonic epilepsy
Source: Epilepsia. 2013 Oct 18;54(12):2158–65. doi: 10.1111/epi.12413 (PMC4209120; doi:10.1111/epi.12413)

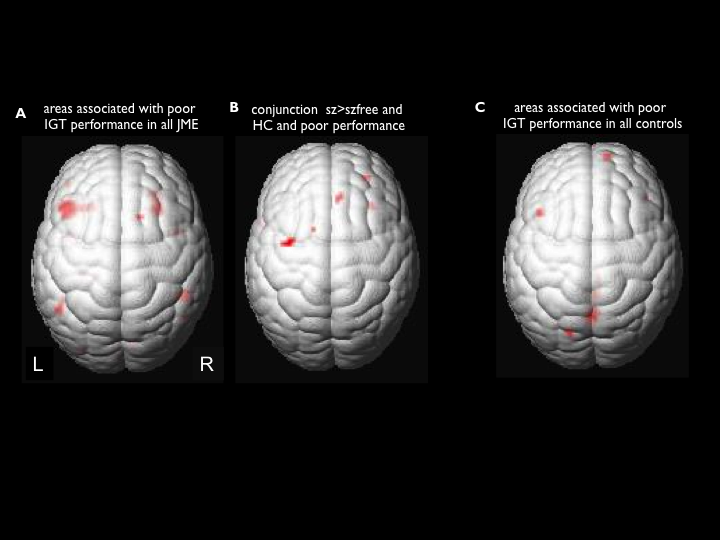

Supplement: Supplementary file 1 — Figure S1. Association of working memory networks activation with IGT performance. [file epi0054-2158-sd1.tif]
