# Supplementary material for: Risk-taking behavior in juvenile myoclonic epilepsy
Source: Epilepsia. 2013 Oct 18;54(12):2158–65. doi: 10.1111/epi.12413 (PMC4209120; doi:10.1111/epi.12413)
Supplement: Supplementary file 2 — Table S1. Clinical details. [file epi0054-2158-sd2.doc]

Supplemantary Table 2. Neuropsychological test results in patients (learners vs. non-learners)

| cognitive abilities | learners | non-learners | statistical analysis* | |
| --- | --- | --- | --- | --- |
|  | Median (range) | Median (range) | U | p |
| *Psychomotor speed*  Trail Making Test A (time in seconds) | 28 (18- 50) | 28 (23-37) | 35.000 | .602 |
| *Mental flexibility*  Trail Making Test time B-A | 31 (5- 62) | 36 (19-57) | 37.000 | .898 |
| *Verbal fluency*  categorial fluency  letter fluency | 19.5 (12.33- 24.0)  15.66 (9.0- 21.66) | 19.17 (12.33- 22.0)  13.83 (10.0- 16.0) | 34.500  28.500 | .547  .274 |
| *Working memory*  subtest Digit Span of the WAIS-III | 20 (9 - 28) | 18 (14- 21) | 26.500 | .282 |

*The Mann-Whitney U Test was applied for behavioural measures.
