# Supplementary material for: Risk-taking behavior in juvenile myoclonic epilepsy
Source: Epilepsia. 2013 Oct 18;54(12):2158–65. doi: 10.1111/epi.12413 (PMC4209120; doi:10.1111/epi.12413)
Supplement: Supplementary file 3 — Table S2. Neuropsychological test results in patients (learners vs. nonlearners). [file epi0054-2158-sd3.docx]

Supplementary data, Figure 1.

Association of working memory networks activation with IGT performance. Overall net scores of IGT performance (C+D – [A+B]) were entered as a covariate. In addition, performance during the 2 Dot back fMRI working memory task was entered as covariate of no interest. Findings demonstrated here are similar to those in figure 2. (A) Negative correlation of IGT-performance with the “2-back minus 0-back”-contrast across the whole JME group revealed bilateral prefrontal cortex activation. (B) Conjunction analysis of JME patients with seizures above seizure-free patients and controls, and negative correlation with IGT-performance (as expressed by overall net scores) revealed hyperactivity in the left DLPFC. The contrast was masked by working memory network activations of healthy controls (p<.05 unc.). (C) In controls, negative correlation of IGT-performance with the “2-back minus 0-back”-contrast across the whole group revealed reduced deactivation of parts of the default mode network, i.e. precuneus, posterior cingulate gyrus and medial prefrontal cortex. (p<.005 unc.)

(CTR= controls, DB= Dot Back, IGT= Iowa Gambling Task, L= left, R= right)
