# Supplementary material for: Risk-taking behavior in juvenile myoclonic epilepsy
Source: Epilepsia. 2013 Oct 18;54(12):2158–65. doi: 10.1111/epi.12413 (PMC4209120; doi:10.1111/epi.12413)
Supplement: Supplementary file 4 [file epi0054-2158-sd4.doc]

*** = non-learner**

| Patient No | time since last seizure  (days) | Seizure type/month | 1.AED, dose/day | 2.AED, dose/day | 3.AED, dose/day |
| --- | --- | --- | --- | --- | --- |
| 1 | 245 | - 1. absence  1. myoclonic jerk | VPA,  800mg | LEV, 1000mg | - |
| 2 | 30 | 1 absence | VPA, 2000mg | LEV, 2000mg | - |
| **3*** | 100 | 0.1 GTCS | VPA, 800mg | LEV, 1000mg | - |
| **4*** | 200 | 0.5 myoclonic jerks 0.2 GTCS | VPA, 1000mg | - | - |
| **5*** | 3 | 4 myoclonic jerks, 1 GTCS | VPA, 2000mg | LEV, 1500mg | - |
| 6 | 14 | 0.4 GTCS | LEV, 1500mg | LTG, 600mg | - |
| **7*** | 0.1 | 600 myoclonic jerks, 4 GTCS | OXC, 2100mg | CLB, 25mg | - |
| 8 | 480 | - | LEV , 250mg | LTG, 400mg | - |
| **9*** | 1800 | - | VPA, 2200mg | - | - |
| 10 | 400 | - | VPA, 2000mg | LEV, 1000mg | LTG, 100mg |
| 11 | 4000 | - | VPA,  200mg | LTG, 400mg | - |
| 12 | 3900 | - | VPA,  600mg | - | - |
| 13 | 2200 | - | - | - | - |
| 14 | 750 | - | VPA, 2000mg | LTG, 150mg | - |
| 15 | 365 | - | PHT,  300mg | - | - |
| 16 | 4500 | - | LTG, 300mg | - | - |
| 17 | 880 | - | VPA, 1000mg | LEV, 1000mg | - |
| 18 | 1200 | - | LEV, 2000mg | - | - |
| 19 | 400 | - | LTG, 200mg | - | - |
| **20*** | 4400 | - | VPA, 1000mg | - | - |
| 21 | 1500 | - | VPA, 2000mg | - | - |

CLB= clobazam

Table 1. Clinical information

GTCS = generalised tonic clonic seizures

LEV = levetiracetam

L TG = lamotrigine

OXC = oxcarbazepine

PHT = phenytoin

VPA = sodium valproate
