# Supplementary material for: Risk-taking behavior in juvenile myoclonic epilepsy
Source: Epilepsia. 2013 Oct 18;54(12):2158–65. doi: 10.1111/epi.12413 (PMC4209120; doi:10.1111/epi.12413)
Supplement: Supplementary file 5 [file epi0054-2158-sd5.pptx]

## Slide 1
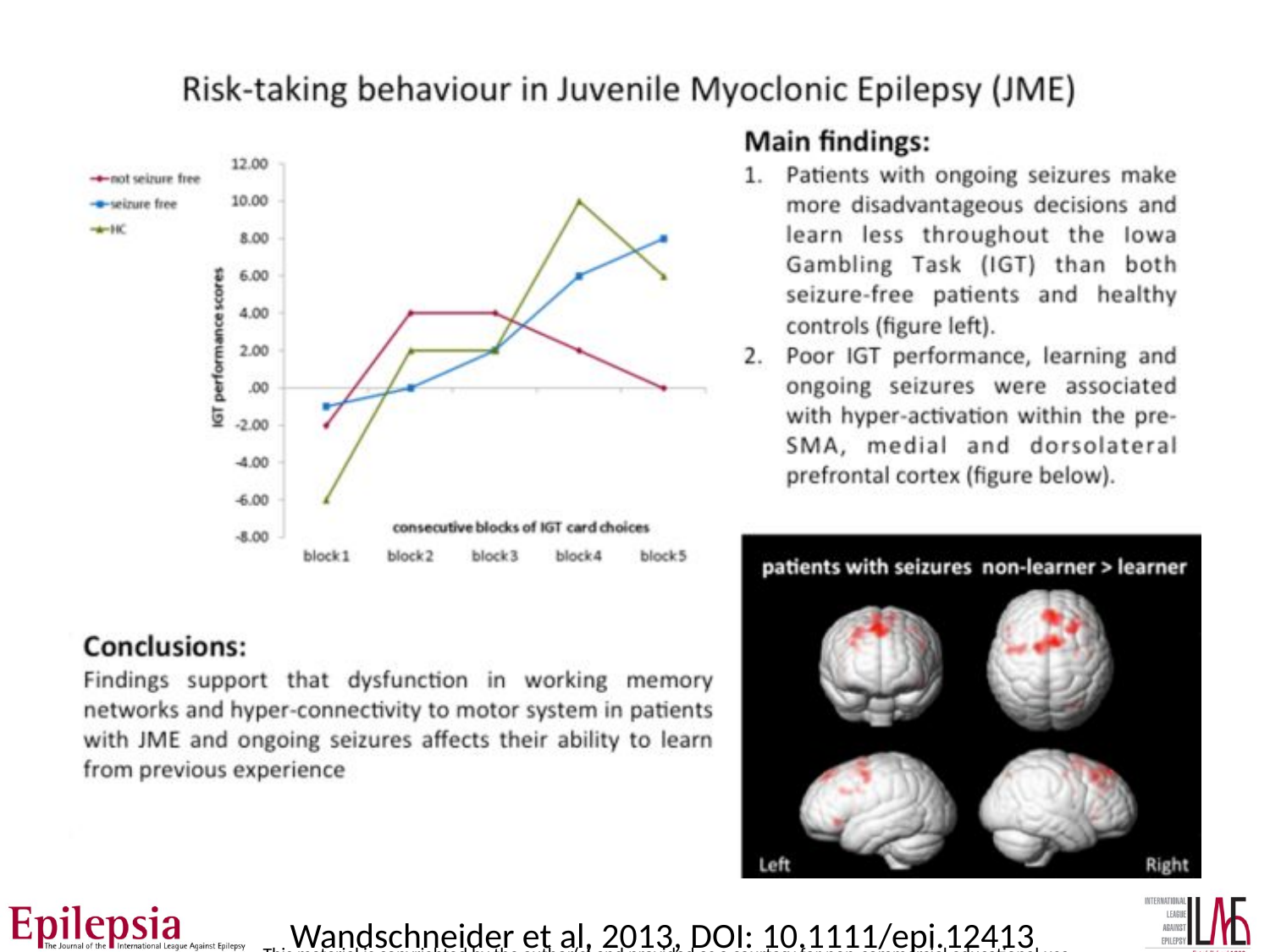

Wandschneider et al, 2013, DOI: 10.1111/epi.12413
This material is copyrighted by the author(s) and provided as a courtesy for non-commercial educational use
